# Recent progress on action and regulation of anorexigenic adipokine leptin

**DOI:** 10.3389/fendo.2023.1172060

**Published:** 2023-07-20

**Authors:** Tadashi Nakagawa, Toru Hosoi

**Affiliations:** ^1^ Department of Clinical Pharmacology, Faculty of Pharmaceutical Sciences, Sanyo-Onoda City University, Sanyo-Onoda, Yamaguchi, Japan; ^2^ Division of Cell Proliferation, ART, Graduate School of Medicine, Tohoku University, Sendai, Miyagi, Japan

**Keywords:** leptin, obesity, mouse model, adipocyte, brain

## Abstract

Organismal energy balance is controlled by inter-tissue communication mediated by the nervous system and hormones, the disruption of which causes metabolic syndrome exemplified by diabetes and obesity. Fat-storing adipose tissue, especially those located in subcutaneous white adipose tissue, secretes leptin in a proportion of fat mass, inhibiting the accumulation of organismal fat by suppressing appetite and promoting energy expenditure. With a prevalence of obesity that exhibits hyperleptinemia, most of the investigation on leptin has been focused on how it works and how it does not, which is expected to be a clue for treating obesity. In contrast, how it is synthesized, transported, and excreted, all of which are relevant to the homeostasis of blood leptin concentration, are not much understood. Of note, acute leptin reduction after hyperleptinemia in the context of obesity exhibited a beneficial effect on obesity and insulin sensitivity, indicating that manipulation of circulating leptin level may provide a therapeutic strategy. Technological advances such as “omics” analysis combined with sophisticated gene-engineered mice studies in the past decade enabled a deeper understanding of leptin’s action in more detail. Here, we summarize the updated understanding of the action as well as regulation of leptin and point out the emerging direction of research on leptin.

## Introduction

Obesity is associated with several metabolic syndromes and its rapid increase in developed countries is now a public concern (1). Obesity is defined by the value of body mass index (BMI > 30) which is calculated by body weight divided by the square of a person’s height. Although fat accumulation in the adipocytes is an end phenotype of this disease, a genome-wide association study of more than 300,000 individuals identified multiple BMI-associated loci that affect gene expression primarily in the neurons (2). In addition, the classic study showed that the lesion in the hypothalamus causes obesity in mice (3). These data suggest that neurons in the hypothalamus are responsible for the pathogenesis of obesity.

In both human beings and mice, the maintenance of organismal fat equilibrium relies upon two distinct types of adipose tissues: white adipose tissue (WAT), responsible for fat uptake and release as needed, and brown adipose tissue (BAT), which utilizes fat for heat generation (4–7). The distribution of BAT is relatively limited, whereas WAT is dispersed throughout the body, categorized into two main types: subcutaneous (sWAT) and visceral (vWAT). While sWAT serves as the primary site for fat storage, vWAT also accumulates fat when an excess amount is present in the body. Emerging evidence further elucidates the functional heterogeneity of adipose tissue determined by its anatomical localization (8).

Leptin is a hormone secreted from the adipocyte, circulates in the bloodstream, and acts on the neurons in the hypothalamus and other brain regions, leading to the inhibition of appetite, enhancement of energy expenditure through activating BAT, and lipolysis in WAT (9). Therefore, leptin deficiency in humans and mice causes obesity through the disruption of these processes (10, 11). Clinical studies showed that the administration of recombinant human leptin or leptin analog (metreleptin) reduced BMI in subgroups of obese adults, revealing that obese patients with high leptin in their blood may not efficiently respond to exogenous leptin (12–14). Therefore, a deeper understanding of leptin regulation and action is necessary to develop leptin-based therapeutics against obesity.

This review overviews the recent advancement of leptin regulation and action, especially focusing on the “omics” analyses and state-of-the-art gene-engineered mice studies. Finally, how knowledge of leptin regulation and action can be translated to the development of anti-obese therapy will be discussed.

## Leptin synthesis and secretion

**Figure 1:**
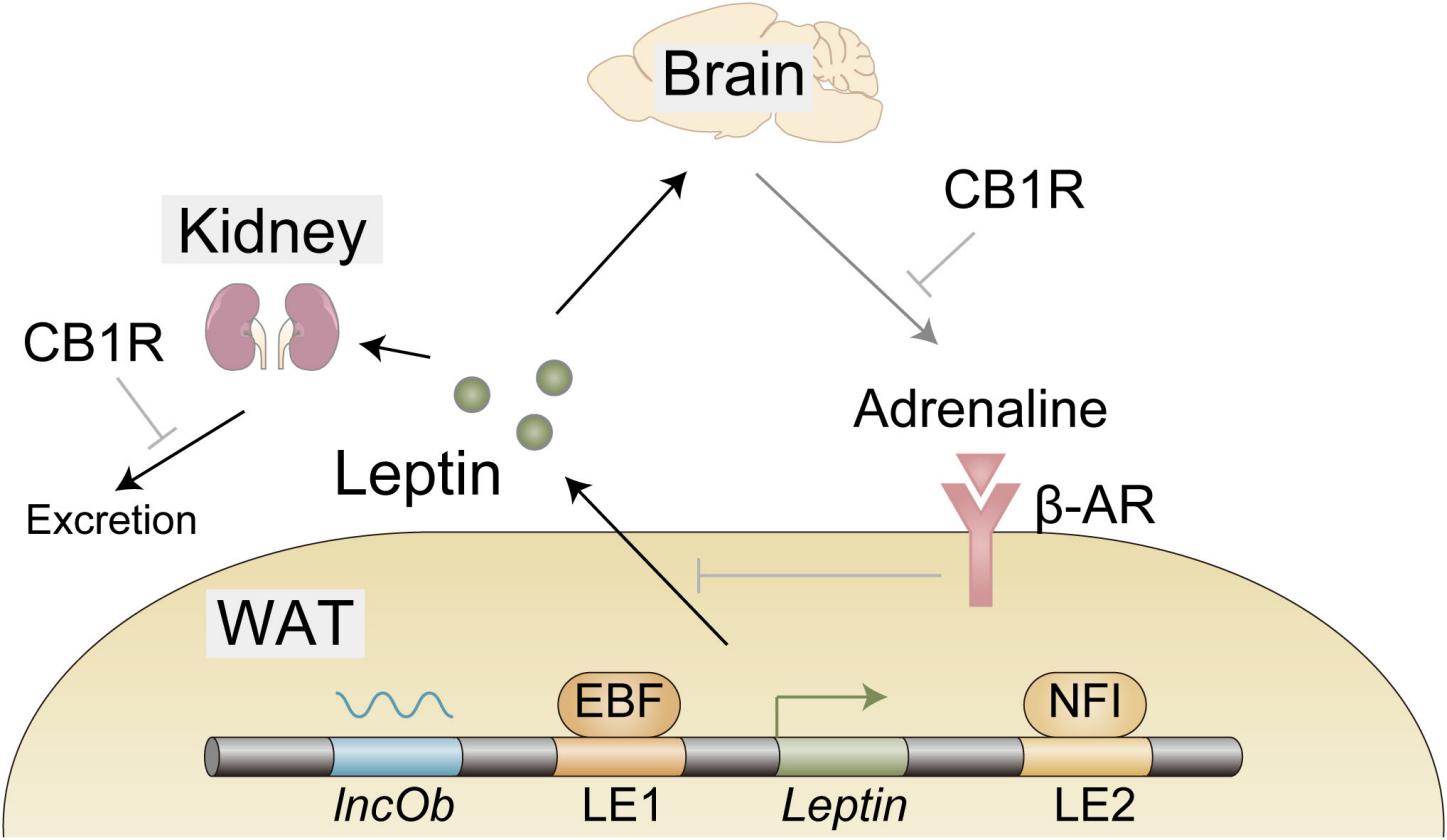
Expression, secretion, and excretion of leptin. EBF and NFI as well as long noncoding RNA *lncOb* cooperatively induce *leptin* gene expression in the white adipose tissue (WAT). Leptin secretion from WAT is negatively regulated by β-adrenergic receptors activated by efferent signals from the brain in response to leptin. A large proportion of leptin is excreted from the kidney. Cannabinoid-1 receptor (CB1R) inverse agonist reduces leptin secretion possibly through reduction of sympathetic tone that is indicated by a decrease in adrenaline in WAT. CB1R inverse agonist also promotes leptin excretion, indicating the involvement of the endocannabinoid system in these processes.

Gene expression is regulated by transcription factors that associate with open chromatin regions called enhancers (15). Assessment of *in vivo* enhancer activity with leptin-bac luciferase mice and genome-wide analysis of open chromatin regions identified two loci that contribute to adipocyte selective expression of leptin - LE1 (located at −16.5 to −16.1 kb upstream of the leptin transcription start site) and LE2 (located at +13.6 to +13.9 kb downstream of the leptin transcription start site) (**Figure 1**) (16, 17). Unbiased proteomic analysis to identify proteins that bind to these regions uncovered retinoid X receptor alpha (RXRα), nuclear factor I (NFI), and early B cell factor (EBF) that are critical for leptin expression (**Figure 1**) (17). CCAAT/enhancer binding protein α (C/EBPα) and specificity protein 1 (SP1) (18), FOS like 2 (FOSL2) (19), nuclear transcription factor Y (NFY) (20), and early growth response 1 (EGR1) induced by insulin (21) have been also reported to play a role in leptin expression and how these factors collaborate with each other to achieve optimal expression of leptin remains to be investigated. Interestingly, Dallner et al. identified long non-coding RNA, named *LncOb*, transcribed from further upstream of the leptin transcription start site (–28 kb) than LE1, that recruits RNA-binding proteins to *leptin* gene promoter to increase *leptin* expression (**Figure 1**) (17). How these RNA-binding proteins regulate leptin expression is not clarified.

As anticipated, leptin levels exhibit elevation during the wakeful phase when organisms consume nutrients and increase fat accumulation in the body. Furthermore, circadian proteins have been also demonstrated to actively participate in the oscillation of leptin expression. Consequently, the elimination of the circadian master gene *Bmal1* specifically in adipocytes in mice disrupts the nocturnal surge of leptin expression and the diurnal decline. Mechanistically, it has been revealed that heightened levels of BMAL1 during the daytime compete with C/EBPα for binding to the leptin promoter, thereby exerting a regulatory influence (22).

Regarding the various types of WAT, it is worth noting that leptin mRNA levels and secretion are twofold higher in subcutaneous sWAT compared to vWAT (23). Given the larger size of sWAT compared to vWAT, it is conceivable that fat mass may influence the expression of leptin levels (23), although the underlying mechanisms remain poorly understood.

Leptin secretion from the adipocytes is negatively regulated by the WAT-innervating sympathetic nervous system (SNS) (24). Since this SNS is activated by leptin stimulation in the hypothalamus (25, 26), it seems likely that leptin downregulates its own secretion *via* the neuro-adipose axis (**Figure 1**). HFD was shown to reduce adrenaline in WAT (27), which might contribute to hyperleptinemia by boosting leptin secretion. Importantly, this reduction of adrenaline is mitigated by the peripheral cannabinoid-1 receptor (CB1R) inverse agonist (27), providing the endocannabinoid system as a therapeutic target as described below (**Figure 1**).

## Leptin excretion

Leptin removal from the circulation is mediated by glomerular filtration in the kidney (28). The following tubular uptake and metabolism of leptin are mediated by endocytic receptor megalin (29, 30). The efficacy of these processes is reduced in high-fat diet (HFD)-induced obese (DIO) mice, indicating that impaired leptin removal, at least in part, contributes to hyperleptinemia (27, 31). To make matters worse, as hyperleptinemia contributes to the pathogenesis of chronic kidney disease (CKD) (32, 33), hyperleptinemia further exacerbates hyperleptinemia by impairing leptin excretion, forming adverse positive feedback to increase blood leptin levels. From the therapeutic point, the endocannabinoid system may be useful, since the CB1R inverse agonist not only increases adrenaline in WAT, but also promotes glomerular filtration and increases megalin expression, resulting in the decreased leptin level in DIO mice (27).

## Leptin transport to the brain parenchyma

Primary sites of leptin action localize in the brain as discussed below. Therefore, leptin needs to be transported to the central nervous system. It is reported that specialized hypothalamic glia named tanycytes internalize blood-borne leptin and release it to CSF, enabling the leptin to reach its sites of action (34). Previously, one variant of the leptin receptors (LepR), ObRa, was suggested to be a transporter of leptin (35, 36), which is supported by a decreased ratio of CSF/plasma leptin level in mice with gene deletion of ObRa (37). Therefore, the involvement of ObRa in tanycytes in leptin transport would be worth investigating. The extent to which hypothalamic glia- and choroid plexus-mediated transport contributes to CSF leptin level awaits to be examined.

## Leptin action – receptor system

There are at least six variants of LepR (38) - ObRa to ObRf - among which ObRa is the most abundantly expressed in the brain except for the hypothalamus where ObRb is dominant (39). The fact that ObRb-specific mutant mice exhibit almost identical phenotypes to those with null mutations of all the variants indicates that ObRb is critical for leptin action (40, 41). In line with this assumption, ObRa-specific knockout mice only manifest a small increase in body weight only when fed an HFD (40).

The functionality of ObRb in energy homeostasis is attributable to its tyrosine phosphorylation sites allowing for the activation of the Janus kinase 2-signal transducer and activator of transcription 3 (JAK2–STAT3) signal transduction pathway (9, 38, 42). Peripheral leptin administration induces rapid increases in phosphorylated STAT3 in several brain regions such as the hypothalamus and brain stem (43), indicating that those regions are responsible for leptin action in maintaining energy balance.

## Leptin action – neural networks

**Figure 2:**
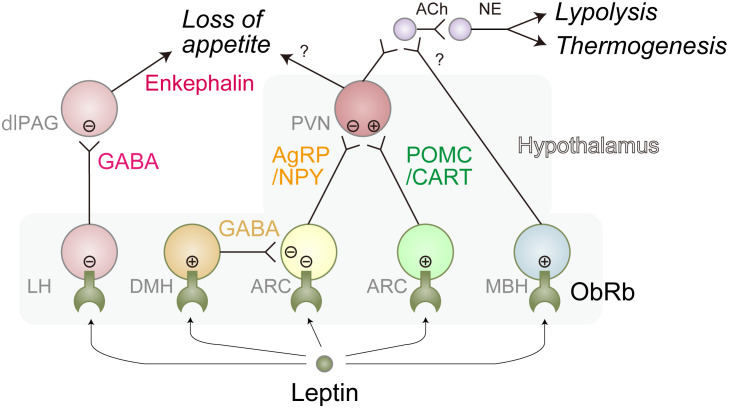
Representative leptin-responsible hypothalamic neurons. Leptin induces loss of appetite, lipolysis, and thermogenesis primarily through these neural networks. There might be more complex interactions between these neurons. Leptin-induced activation and inhibition are represented as circled plus mark and circled minus mark, respectively. dlPAG, dorsolateral periaqueductal gray; LH, lateral hypothalamus; ARC, arcuate nucleus; PVN, paraventricular nucleus; MBH, mediobasal hypothalamus; Ach, acetylcholine; NE, norepinephrine; GABA, Gamma-aminobutyric acid; AgRP, agouti-related peptide; NPY, neuropeptide Y; POMC, proopiomelanocortin; CART, Cocaine- and amphetamine-regulated transcript. Of note, ARC is a part of MBH.

The motivation to eat is driven by neural networks which can be functionally divided into three sub-modules; the autonomic module that senses nutritional or energy reserve status in the organism, the reward module that establishes the “liking” or “wanting” properties of eating-related stimuli, and the executive module responsible for the decision to eat (44). The best-characterized function of leptin is related to the autonomic module in which it inactivates Agouti-related peptide (AgRP)/Neuropeptide Y (NPY) neurons, while it stimulates Pro-opiomelanocortin (POMC)/Cocaine- and amphetamine-regulated transcript (CART) neurons, resulting in the reduction of appetite as well as increases in locomotion, thermogenesis, and lipolysis (9, 38, 42). Deletion of LepR gene specifically in AgRP/NPY, but not in POMC/CART, neurons in the adult mice (to avoid compensatory effects of gene deletion) causes obesity under a standard chow diet (45–47). On the other hand, loss of LepR in POMC/CART, but not AgRP/NPY, neurons in the adult mice promotes obesity only when fed HFD (43–45) These results suggest that the primary target of leptin is context-dependent. Consistently, a recent report shows that fatty acids are involved in the activation of POMC/CART neurons (48). In addition, leptin was demonstrated to activate ventral dorsomedial hypothalamus (vDHM)-located Gamma-aminobutyric acid (GABA) neurons that inactivate AgRP/NPY neurons (45) and mediobasal hypothalamus (MBH)-located SH2B-expressing neurons that increase the tone of SNS as described below (49). Furthermore, it negatively controls lateral hypothalamus (LH)-localized GABA neurons that inactivate Proenkephalin (Penk)-expressing dorsolateral periaqueductal gray (dlPAG) neurons, leading to increased level of food intake (50) (**Figure 2**). Of note, selective deletion of LepR in LH neurons in adult mice causes obesity only under HFD conditions, reinforcing the notion that the primary target of leptin is context-dependent (50).

The thermogenic and lipolytic effects of leptin are thought to be largely mediated through BAT and WAT-innervating SNS, respectively, as physical or genetic denervation suppresses leptin-induced lipolysis gene expression in WAT (25) and thermogenesis in BAT (51). Leptin positively regulates these SNS neurons through MBH-localized AgRP/NPY-, POMC/CART-, and SH2B-expressing neurons since deletion of LepR in those neurons results in obesity with the inefficiency of thermogenesis and lipolysis (**Figure 2**) (45–47).

Besides the autonomic module, it is also reported that leptin directly acts on striatal dopamine (DA) neurons involved in the reward module (52, 53). Recently, it is revealed that LepR-expressing striatal cholinergic interneurons (ChIs) mediate striatal DA release by leptin (54).

## Therapies acting on leptin function as anti-obesity drugs

Leptin has a profound effect on the reduction of body weight in obese patients who exhibit a low level of circulating leptin; designated as type 1 obesity (14). However, its effect is barely detectable in people with hyperleptinemia (type 2 obesity), limiting the utility of leptin as an anti-obesity drug (14). Since type 2 obesity accounts for the great majority of obese patients, leptin resistance needs to be overcome. To this end, recovery of leptin sensitivity or bypass of leptin signaling would be two main strategies.

To find leptin sensitizers, it is critical to understand how the response to leptin is diminished in LepR-expressing neurons in the setting of obesity. Utilizing ObRb-expressing cultured neurons we established to circumvent the heterogeneity of tissue samples, we reported that endoplasmic reticulum (ER) stress which is known to be activated under the obese condition inhibits leptin signaling through protein tyrosine phosphatase non-receptor type 1 (PTPN1, also known as PTP1B) that dephosphorylates leptin-induced phospho-JAK2 (55). The negative effects of ER stress on leptin signaling in the LepR-expressing neurons in mouse brains were also reported by other research groups (56, 57), reinforcing the notion that mitigating ER stress can lead to leptin sensitization. Our subsequent studies identified fluvoxamine (58), flurbiprofen (59), caffeine (60), and biochanin A (61) that attenuate ER stress and enhance leptin signaling, providing the evidence that these compounds are potential therapeutic drugs. Other candidates targeting ER stress include 4-phenyl butylate and tauroursodeoxycholic acid (56), celastrol (62), and withaferin A (63).

As phosphorylation-induced activation of JAK/STAT plays an essential role in LepR signaling, reduction of JAK/STAT attenuator might be effective to augment leptin sensitivity. In addition to PTPN1 which inhibits JAK2 activation as mentioned above, protein tyrosine phosphatase non-receptor type 2 (PTPN2, also known as TCPTP) inhibits leptin signaling by dephosphorylating STAT3 (64). *In vivo* significance of these negative regulators in leptin resistance was demonstrated by combined deletions of *Ptpn1/ptpn2* genes in adult obese mice that reinstated the leptin signaling (65). Importantly, simultaneous inhibition of PTPN1 and PTPN2 by intranasal administration of RU486 and claramine rescues leptin sensitivity in obese mice (65), suggesting that these phosphatases are therapeutic targets, and intranasal administration could circumvent the transport barrier of drugs across the BBB to the hypothalamus.

In contrast to JAK/STAT attenuators, JAK/STAT activators might be harnessed as leptin sensitizers. Support for this strategy was provided by overexpression of a potent JAK2 activator SH2B adaptor protein 1 (SH2B1) in the hypothalamus (49), as well as overexpression of LepR activator growth factor receptor bound protein 10 (Grb10) in the AgRP/NPY and POMC/CART neurons (47), both of which protected against obesity in DIO mice

ObRb leptin receptor is subjected to inactivation in obese conditions as exemplified by the cleavage of its leptin-binding regions in the extracellular domain by matrix metalloprotease 2 which is secreted by astrocytes and AgRP neurons under the conditions of obesity (66). Therefore, a forced increase in the ObRb levels might not improve leptin resistance.

Counterintuitively, emerging evidence indicates that partial leptin reduction serves as a leptin sensitizer (67), based on the finding that transgenic overexpression of leptin in adult DIO mice paradoxically led to weight gain (68). Consistently, administration of leptin antibody and partial deletion of *leptin* genes (68), CB1R inverse agonist (30), and auranofin (69) in adult DIO mice, all of which achieve a partial reduction of circulating leptin levels, lead to body weight reduction in a manner dependent on intact leptin signaling. Even without any manipulation, time-restricted feeding was recently shown to reduce leptin expression (70), providing a feasible means to reduce leptin levels. Whether these compounds or methods can be applied to human obese patients awaits further investigation with emphasis on side effects.

Several studies have documented the impact of dietary composition on leptin activity in humans (71). As an illustration, a high-protein diet has been shown to decrease appetite and body weight without inducing leptin, thereby indicating enhanced leptin sensitivity (72). Omega-3 fatty acids have also been observed to lower fat mass with increased or unchanged leptin levels in obese individuals, possibly due to partial reduction-induced recovery of leptin action (73, 74). These findings suggest the possibility of regulating leptin activity by monitoring dietary intake.

The caveat to the reduction of leptin levels is also raised. Lipectomy represents the most uncomplicated approach to reducing adipose tissue. However, the consequential reduction in leptin levels is postulated to underlie the phenomenon of weight regain (75), highlighting the necessity of maintaining adequate levels of leptin following acute adiposity loss. Whichever it is, the utilization of interventions that modulate leptin levels may potentially disrupt the hormonal equilibrium of the organism, thereby posing a potential hazard of unanticipated adverse reactions. Hence, meticulous deliberation is warranted when contemplating the administration of such agents.

Bypassing leptin signaling through reducing fat mass and enhancing thermogenesis was successful in mouse models in which WAT-innervating sympathetic neurons are optogenetically activated (25) and BAT-innervating neurons are genetically increased (49), respectively. These findings may be the basis of the future development of leptin-bypassing therapy for obesity.

## Author contributions

Conceptualization, Literature research, Draft preparation: TN and TH. All authors contributed to the article and approved the submitted version.
